# Accelerated telomere shortening in adrenal zona reticularis in patients with prolonged critical illness

**DOI:** 10.3389/fendo.2023.1244553

**Published:** 2023-09-04

**Authors:** Keisuke Nonaka, Kaiyo Takubo, Junko Aida, Yoriko Watai, Akiko Komatsu, Fujiya Gomi, Yuuki Shichi, Yuto Yamazaki, Toshiyuki Ishiwata, Hironobu Sasano, Tomio Arai

**Affiliations:** ^1^ Division of Aging and Carcinogenesis, Research Team for Geriatric Pathology, Tokyo Metropolitan Institute for Geriatrics and Gerontology, Tokyo, Japan; ^2^ Department of Drug Discovery Support Business, Summit Pharmaceuticals International, Tokyo, Japan; ^3^ Department of Pathology, Tokyo Metropolitan Institute for Geriatrics and Gerontology, Tokyo, Japan; ^4^ Department of Pathology, Tohoku University Graduate School of Medicine, Sendai, Japan

**Keywords:** telomere, adrenal gland, zona reticularis, prolonged critical illness, DHEA, hypoalbuminemia

## Abstract

**Background:**

The number of patients with prolonged critical illness (PCI) has been increasing in many countries, and the adrenal gland plays an important role in maintaining homeostasis during PCI. Chronic disease burden is reportedly associated with shorter telomere lengths in human tissues. Telomere shortening in human somatic cells is largely dependent on cell divisions, and critically short telomeres lead to cellular dysfunction and aging. However, the association between PCI and telomere lengths in human adrenal cells is poorly understood. In this study, we investigated this association to assess whether the burden of PCI could accelerate the aging process in adrenal cells.

**Methods:**

Adrenocortical tissues from patients who died after PCI usually show a diffuse pattern of intracellular cholesterol ester depletion (i.e., lipid depletion). This study examined near-normal adrenal glands obtained from autopsied patients who died suddenly (control group) and lipid-depleted adrenal glands obtained from autopsied patients who died after PCI (PCI group). The control group included 7 men aged 80 to 94 years (mean age: 85.3 years) and 7 women aged 84 to 94 years (mean age: 87.7 years). The PCI group included 10 men aged 71 to 88 years (mean age: 78.8 years) and 8 women aged 77 to 95 years (mean age: 85.6 years). By using quantitative fluorescence *in situ* hybridization, relative telomere lengths (RTLs) were determined in the parenchymal cells of the three adrenocortical zones (zona glomerulosa, zona fasciculata, and zona reticularis [ZR]) and in the chromaffin cells of the medulla. The number of adrenal parenchymal cells was determined by immunohistochemistry and digital image analysis.

**Results:**

RTLs in ZR cells were significantly shorter in the PCI group than in the control group for both men and women (*P* = 0.0001 for men and *P* = 0.0012 for women). However, RTLs in the remaining three types of adrenal cells did not differ between the control and PCI groups for both men and women. The number of ZR cells was higher in the PCI group than in the control group for both men and women (*P* < 0.0001 for both men and women). The proportion of the number of ZR cells to the total number of adrenocortical parenchymal cells was also higher in the PCI group than in the control group (*P* < 0.0001 for both men and women). The Ki-67 proliferation index in ZR cells was higher in the PCI group than in the control group (*P* = 0.0039 for men and *P* = 0.0063 for women).

**Conclusions:**

This study demonstrated ZR cell-specific telomere shortening in patients with adrenal lipid depletion who died after PCI. Our results suggest that the reactive proliferation of ZR cells accelerates the telomere shortening and aging process in ZR cells in these patients. The results of our study may contribute to the understanding of adrenal aging during PCI.

## Introduction

Prolonged critical illness (PCI) is prevalent in the elderly (1, 2). Given that many countries have recently faced a large increase in the elderly population (3), the number of patients with PCI has also increased.

As the end organ of the hypothalamic–pituitary–adrenal axis and sympathetic adrenomedullary system, the human adrenal gland plays an important role in maintaining homeostasis under various conditions, including PCI (4). The adrenal gland consists of two distinct regions: the cortex and medulla. The cortex secretes steroid hormones, whereas the medulla, which predominantly comprises chromaffin cells, secretes catecholamines (5, 6). The human adrenal cortex has three morphologically and functionally defined zones: the zona glomerulosa (ZG) (outermost layer), zona fasciculata (ZF) (middle layer), and zona reticularis (ZR) (innermost layer) (7). The ZG and ZF synthesize mineralocorticoids (particularly aldosterone) and glucocorticoids (particularly cortisol), respectively (8). The ZR synthesizes adrenal androgens such as dehydroepiandrosterone (DHEA) and its sulfate (DHEA-S).

Several studies have reported that chronic disease burden is associated with shorter telomere lengths in human tissues (9, 10). Telomeres are DNA sequences that protect the ends of eukaryotic chromosomes (11, 12). Telomere lengths in human somatic cells shorten with each cell division, a phenomenon known as the end replication problem (12, 13). Critically shortened telomeres trigger a DNA damage response, causing maladaptive cellular changes and cell cycle arrest (12, 14). Therefore, telomere shortening is an important hallmark of cellular aging (15).

However, the association between PCI and telomere lengths in human adrenal cells is poorly understood. This study investigated the association between PCI and telomere lengths in four different types of human adrenal cells (ZG, ZF, and ZR parenchymal cells and adrenomedullary chromaffin cells). We conducted quantitative fluorescence *in situ* hybridization (Q-FISH) to quantify individual telomere and centromere signals at the single-cell scale and measured the relative telomere lengths (RTLs) (telomere signal intensities relative to centromere signal intensities) in four different types of adrenal cells. We aimed to assess whether the burden of PCI could accelerate the aging process in adrenal cells with the goal of improving the treatment of patients with PCI.

## Materials and methods

### Selection of human adrenal tissues

We examined autopsied human adrenal glands. Adrenocortical steroid hormones are synthesized from cholesterol, which is mostly esterified and stored in intracellular lipid droplets until steroidogenesis (16). Adrenocortical tissues obtained from autopsied patients who died after PCI usually show a diffuse pattern of cholesterol ester depletion (hereinafter lipid depletion) (16, 17). In contrast to death from PCI, death occurring within 24 h of symptom onset is referred to as sudden death (18). Adrenocortical tissues obtained from autopsied patients who died suddenly are considered under near-normal physiological conditions. In the current study, we defined the control group as patients who died suddenly and the PCI group as patients with adrenal lipid depletion who died after PCI.

Regarding the PCI group, adrenal lipid depletion can be histologically identified using hematoxylin and eosin (H&E) staining. To obtain adrenal glands with lipid depletion, we assessed the adrenal glands obtained from 262 serial autopsies conducted between September 2015 and December 2020 at the Tokyo Metropolitan Institute for Geriatrics and Gerontology, Tokyo, Japan. Immediately after harvesting during the autopsy, the adrenal glands were routinely fixed with 10% buffered formalin and sectioned at 5-mm intervals in a plane vertical to the long axis. Each section was processed for microscopic examination. A 2-μm-thick section was cut from each paraffin-embedded tissue block and stained with H&E. Three pathologists (K. N., Y. Y., and H. S.) examined H&E-stained sections independently and selected 26 patients with adrenal lipid depletion. The exclusion criteria were as follows: (i) a history of endocrine disorders such as pituitary adenomas and adrenocortical disorders; (ii) use of any form of corticosteroids or drugs that possibly affect the hypothalamic–pituitary–adrenal axis, at least during the last month before death; (iii) primary or metastatic adrenal tumors; and (iv) diffuse necrosis, autolysis, or hemorrhage in the adrenal tissue. In accordance with these criteria, eight patients were withdrawn from this study. Overall, the PCI group included 18 elderly patients with adrenal lipid depletion (10 men and 8 women). All patients in the PCI group were Japanese and died of prolonged critical illness.

Regarding the control group, 18 age- and sex-matched specimens of autopsied patients who died a sudden death (from January 2002 to December 2020) were retrieved from the pathology files of the Tokyo Metropolitan Institute for Geriatrics and Gerontology, Tokyo, Japan. Adrenal H&E-stained sections (cut in a central plane vertical to the long axis) were examined independently by three pathologists (K. N., Y. Y., and H. S.), and four patients were withdrawn from the study (based on the exclusion criteria). Adrenal lipid depletion was not observed in any of these patients. Overall, the control group included 14 elderly patients with adrenals showing near-normal conditions (7 men and 7 women). All patients in the control group were Japanese.

### RTLs measurement using Q-FISH

Ten percent formalin-fixed paraffin-embedded (FFPE) samples of left or right adrenals were sliced into 2-μm sections for determination of telomere length using Q-FISH. The tissue slides were processed by the Q-FISH method, as described previously (19, 20). Tissue sections were hybridized with peptide nucleic acid probes for the telomere (Telo C-Cy3 probe: 5′-CCCTAACCCTAACCCTAA-3′; catalogue number F1002, Fasmac, Atsugi, Japan) and centromere (Cenp1-fluorescein isothiocyanate or FITC probe: 5′-CTTCGTTGGAAACGGGGT-3′; custom-made, Fasmac), and the nuclei were stained with 40,6-diamidino-2-phenylindole (DAPI) (Molecular Probes, Eugene, OR, USA). In Q-FISH images, we could identify the individual ZG and ZF parenchymal cells and chromaffin cells, based on their respective morphological features: ZG cells had less abundant cytoplasm compared to ZF cells, which were organized into bundles, and chromaffin cells had relatively regular nuclei in size and contour (21–23). ZR parenchymal cells could be identified because they had cytoplasm with prominent lipofuscin, displaying brilliant yellow autofluorescence in Q-FISH images (24). Q-FISH digital images were captured using a charge-coupled device camera (RETIGA R3; Teledyne Photometrics, Surrey, BC, Canada) mounted on an epifluorescence microscope (80i, Nikon, Tokyo, Japan) equipped with a triple band-pass filter set for DAPI/fluorescein isothiocyanate (FITC)/Cy3 (Part #61010, Chroma Technology Corp., Rockingham, VT, USA) and a 40× objective lens (Plan Fluor 40/0.75; Nikon). Microscopic control and image acquisition were performed using the Image-Pro Plus software package (version 7.0; Media Cybernetics Co. Ltd., Silver Spring, MD, USA). As reported previously, the captured images were analyzed with our original tissue analysis software, TissueTelo, which can calculate the telomere to centromere ratio (TCR) as an estimate of the relative telomere length in the individual nucleus (19, 25). As a control for variations during sample preparation, we also performed Q-FISH in a cell block-section of a cultured cell line, TIG-1 (26), placed on the same slides as the adrenal gland sections. TIG-1 is a human fibroblast strain established from fetal lung and commonly used in studies of cellular senescence (26, 27). The TIG-1 cell line was subcultured (34 PDL; population doubling level) and had a telomere length of 8.64 kbp, as measured by Southern blot analysis. Each TCR for the target cells was divided by the mean TCR for the cell block on the same slide to obtain the normalized TCR (NTCR). We measured the NTCR of individual parenchymal cells in the three adrenocortical zones and the adrenomedullary chromaffin cells. In each type of adrenal parenchymal cells of each patient, the number of cells analyzed ranged from 110–120, and we defined the mean NTCR as RTL in this study.

### Measurement of the number of adrenocortical parenchymal cells

#### Immunohistochemistry

Cytochrome B5 (CytB5), an accessory protein important in the regulation of 17,20-lyase activity, is exclusively expressed in ZR parenchymal cells (28), but not in the other three types of adrenal cells (i.e., the ZG and ZF cells and the adrenomedullary chromaffin cells). Immunohistochemical analysis of CytB5 was performed to identify ZR parenchymal cells. Immunohistochemical analysis of chromogranin A (CgA) was conducted to distinguish adrenocortical parenchymal cells from adrenomedullary chromaffin cells. Specifically, CgA is a glycoprotein stored in the secretory granules of adrenomedullary chromaffin cells (29). Immunohistochemical staining was performed on thin sections (2 µm) of ten percent FFPE specimens. The samples were deparaffinized with xylene and hydrated using a graded series of ethanol solutions, and then, antigen retrieval was performed using a HEAT PROCESSOR solution (10 mM, sodium citrate buffer, pH 6.0; 715281; Nichirei Biosciences, Tokyo, Japan); the samples were incubated in the solution at 100 °C for 20 min. Next, the sections were incubated with Protein Block-Serum Free (X0909; Dako, California, USA) for 5 min to block nonspecific antibody binding sites and then incubated with the primary antibodies against CytB5 (ab69801, diluted 1:1000, 1 h at RT; Abcam, Shanghai, China). The sections were incubated with 3% H_2_O_2_ solution at RT for 5 min to block endogenous peroxidase activity; subsequently, they were incubated in Histofine Simple Stain MAX PO (MULTI; 724152; Nichirei Biosciences, Tokyo, Japan) for 30 min at RT. The sections were stained using 3,3′-diaminobenzidine (DAB). The specifically bound antibodies were eluted with 0.1 N HCl. Next, the sections were incubated with Protein Block-Serum Free (Dako) for 5 min, and then incubated with the primary antibodies against CgA (712751, Ready-To-Use, 1 h at RT; Nichirei Biosciences). Subsequently, the samples were incubated with secondary antibodies (1:150, goat biotinylated anti-rabbit IgG, BA-1000; Vector Laboratories, Burlingame, CA, USA) for 30 min at RT. The slides were then incubated with the Vectastain ABC-AP reagent (AK-5000; Vector Laboratories) for 30 min; the Vector Red substrate kit (SK-5100; Vector Laboratories) was used to stain the samples, and hematoxylin was used for counterstaining.

#### Digital image analysis

For each patient, three arbitrary digital images of immunohistochemically stained sections were taken at 40× magnification using a Mantra multispectral microscope (PerkinElmer, Waltham, MA, USA). The same camera settings were used to acquire all the images. Each image covered the area from the adrenal capsule to the medulla. After image capture, the fields of view were spectrally unmixed and then analyzed using supervised machine-learning algorithms (available with the inForm 2.4 software; PerkinElmer). The software enabled us to analyze the number of CytB5-positive cells (i.e., ZR parenchymal cells) and total number of adrenocortical parenchymal cells in the individual images. The same threshold for DAB positivity (0.06) was applied to all images. Vector Red staining for CgA (i.e., staining for adrenomedullary chromaffin cells) helped the software recognize adrenocortical parenchymal cells.

### Ki-67 proliferation index assessment in ZR parenchymal cells

#### Immunohistochemistry

Double-staining immunohistochemistry for CytB5 and Ki-67 was performed to investigate the Ki-67 proliferation index (i.e., the percentage of cells with positive nuclear Ki-67 immunostaining) in ZR parenchymal cells. The immunohistochemical staining methods used were the same as those used in the aforementioned double-staining immunohistochemistry for CytB5 and CgA, except that primary antibodies against Ki-67 (M7240, diluted 1:100, 1 h at RT; Dako, CA, USA) and secondary antibodies (MP-5402, Ready-To-Use, 30 min at RT, anti-mouse IgG polymer; Vector Laboratories) were used instead of primary antibodies against CgA and secondary antibodies (BA-1000; Vector Laboratories).

#### Digital image analysis

For each patient, three arbitrary digital images of immunohistochemically stained sections were obtained at 200× magnification using a Mantra multispectral microscope. The same camera settings were used to acquire all the images. After image capture, the fields of view were spectrally unmixed and analyzed using supervised machine-learning algorithms. The same thresholds for DAB positivity (0.2) and Vector Red positivity (0.3) were applied to all images.

### Measurement of the number of adrenocortical cells expressing cytochrome P450 17A1 (CYP17A1)

#### Immunohistochemistry

CytB5 is an allosteric regulator of cytochrome P450 17A1 (CYP17A1), which enhances its 17,20-lyase activity. CYP17A1 catalyzes the synthesis of DHEA from pregnenolone (30). Double-staining immunohistochemistry for CYP17A1 and CgA was performed to identify the cells expressing CYP17A1 in the adrenal cortex. Immunohistochemical analysis of CgA was conducted to distinguish adrenocortical parenchymal cells from adrenomedullary chromaffin cells, as described above. The immunohistochemical staining methods used were the same as those used in the aforementioned double-staining immunohistochemistry for CytB5 and CgA, except that primary antibodies against CYP17A1 (HPA048533, diluted 1:200, 1 h at RT; Atlas Antibodies, Stockholm, Sweden) were used instead of those against CytB5.

#### Digital image analysis

For each patient, three arbitrary digital images of immunohistochemically stained sections were obtained at 100× magnification using a Mantra multispectral microscope. The same camera settings were used to acquire all the images. Each image covered the area from the adrenal capsule to the medulla. After image capture, the fields of view were spectrally unmixed and analyzed using supervised machine-learning algorithms. The same threshold for DAB positivity (0.09) was applied to all the images.

### Antemortem serum DHEA-S levels

At the Tokyo Metropolitan Institute for Geriatrics and Gerontology, residual serum specimens obtained from patients in a hospital ward or emergency department are stored for one week in laboratory refrigerators for additional tests. When the patients die and autopsies are performed, after obtaining informed consent, the residual serum samples are pipetted into several 1.5-mL plastic vials and stored at -80 °C until assayed. In this study, residual serum samples were obtained from five men and five women in the control group, and eight men and five women in the PCI group. The DHEA-S levels were measured by chemiluminescent enzyme immunoassay using a UniCel DxI 800 Access Immunoassay System (Beckman Coulter Inc., Brea, CA, USA).

### Antemortem serum albumin levels

We examined the antemortem serum albumin levels by referring to the medical records of each patient in the control and PCI groups.

### Assessment of oxidative stress and DNA damage in adrenal parenchymal cells

Patients with PCI experience oxidative stress caused by reactive oxygen and nitrogen species (31, 32). Therefore, we performed immunohistochemical analyses of 8-hydroxydeoxyguanosine (8OHdG) and phosphorylated gamma histone H2AX (γH2AX), which are representative markers of oxidative stress and DNA damage (33, 34).

#### Immunohistochemistry for 8OHdG

Immunohistochemical staining was performed on thin sections (2 µm) of ten percent FFPE specimens. The samples were deparaffinized with xylene and hydrated using a graded series of ethanol solutions; then, antigen retrieval was performed using a HEAT PROCESSOR solution (10 mM, sodium citrate buffer, pH 6.0; 715281; Nichirei Biosciences). The samples were incubated in the solution at 100 °C for 20 min. Next, the sections were incubated with Protein Block-Serum Free (X0909; Dako) for 5 min to block nonspecific antibody binding sites and then incubated with primary antibodies against 8OHdG (MOG-020P, diluted 1:100, 1 h at RT; JaICA, Shizuoka, Japan). The sections were incubated with 3% H_2_O_2_ solution at RT for 5 min to block endogenous peroxidase activity and subsequently incubated in Histofine Simple Stain MAX PO (MULTI; 724152; Nichirei Biosciences) for 30 min at RT. The sections were stained using DAB. Hematoxylin was used for counterstaining.

#### Immunohistochemistry for γH2AX

The immunohistochemical staining methods used were the same as those used in the above-mentioned immunohistochemistry for 8OHdG, except that primary antibodies against γH2AX (NB100-384, diluted 1:2000, 1 h at RT; Novus Biologicals, CO, USA) instead of those against 8OHdG.

### Statistical analysis

Statistical analysis was performed using GraphPad Prism version 5.01 for Windows (GraphPad Software Inc., CA, USA). In this study, the Mann–Whitney test was used to compare two groups. When the Mann–Whitney test was not applicable, the Wilcoxon signed–rank test was used to compare the two groups. The Kruskal–Wallis test with Dunn’s multiple comparison test was used to compare three or more groups. Correlations were analyzed using Spearman’s correlation coefficients. Statistical significance was set at *P* < 0.05.

## Results

### Clinicopathological characteristics of autopsied patients

**Figure 1:**
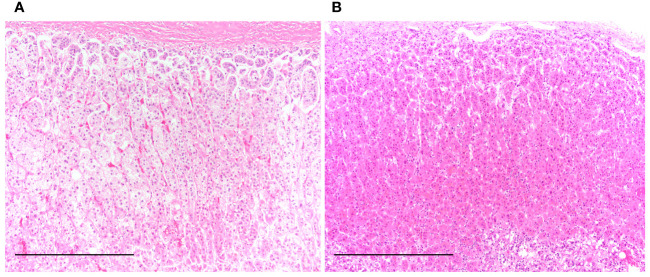
Representative hematoxylin and eosin-stained histological images of the adrenal gland in the control and prolonged critical illness (PCI) groups. **(A)** Clear lipid-laden adrenocortical cells are seen in the near-normal adrenal gland of a patient from the control group. **(B)** Reddish-pink lipid-depleted adrenocortical cells are seen in the adrenal gland of a patient from the PCI group. Scale bar in **(A)** and **(B)**, 500 μm.

**Table 1** summarizes the clinicopathological characteristics of autopsied patients. The mean age in the control group was 85.3 years in men and 87.7 years in women, whereas that in the PCI group was 78.8 years in men and 85.6 years in women. Disease duration represents the estimated period from symptom development to death (**Table 1**). **Figure 1** shows the representative H&E-stained histological images of the adrenal glands in the control and PCI groups.

**Table 1:** Summary of the clinicopathological characteristics of autopsied patients.

Control group							
Men				Women			
#	Age	Disease duration	Pathological findings (causes of death)	#	Age	Disease duration	Pathological findings (causes of death)
1	80	15 h	Common bile duct stones, sepsis	1	84	13 h	Cerebral hemorrhage, cerebral hernia
2	81	12-13 h	Sigmoid colon perforation, acute generalized peritonitis	2	85	12-13 h	Rectal perforation, acute generalized peritonitis
3	83	23-24 h	Hemorrhagic cerebral infarction	3	86	10-11 h	Acute myocardial infarction, cardiac tamponade
4	83	23-24 h	Cerebral hemorrhage, cerebral hernia	4	86	16 h	Ruptured thoracic aortic aneurysm
5	87	12-13 h	Hemorrhagic cerebral infarction, cerebral hernia	5	89	1-2 h	Aspiration pneumonia, food debris in bilateral main bronchi
6	89	2-3 h	(Lethal arrhythmia) Autopsy did not discover the cause of death	6	90	9 h	Acute myocardial infarction, cardiac tamponade
7	94	12-13 h	Cerebral hemorrhage, cerebral hernia	7	94	1 h	Airway obstruction with mucus and food debris
PCI group							
Men				Women			
#	Age	Disease duration	Pathological findings (causes of death)	#	Age	Disease duration	Pathological findings (causes of death)
1	71	1 month	Gastric cancer with multiple metastases	1	77	1 month	Hepatitis C-related liver cirrhosis
2	72	46 days	Fulminant myocarditis	2	78	21 days	Intra-abdominal hemorrhage following pancreaticoduodenectomy
3	73	3 months	Pancreatic cancer with multiple metastases	3	86	5 months	Pancreatic cancer with multiple metastases, bronchopneumonia, diffuse alveolar damage
4	74	7 months	Sigmoid colon cancer, gastrointestinal hemorrhage	4	86	40 days	Bronchopneumonia, lung abscess, bilateral pleural effusions, sepsis
5	74	1 year	Acute myeloid leukemia with myelodysplasia-related changes	5	86	2 months	Diabetic nephropathy, old myocardial infarction, bilateral pleural effusions, pulmonary edema
6	81	1 month	Aspiration pneumonia, lung abscess, sepsis	6	87	2 months	Hepatitis C, idiopathic portal hypertension, ruptured gastric varices
7	81	21 days	Chronic hepatitis, aspiration pneumonia, diffuse alveolar damage	7	90	28 days	Myelodysplastic syndrome, pancytopenia
8	86	3 months	Subcutaneous abscess, sepsis	8	95	1 month	Aortic valve infective endocarditis, acute myocardial infarction
9	88	37 days	Prostate cancer, pyelonephritis, sepsis				
10	88	5 months	Ischemic enteritis, gastrointestinal hemorrhage, aspiration pneumonia				

Disease duration represents the estimated time period from symptom development to death. PCI, Prolonged critical illness.

### RTLs in adrenal parenchymal cells

**Figure 2:**
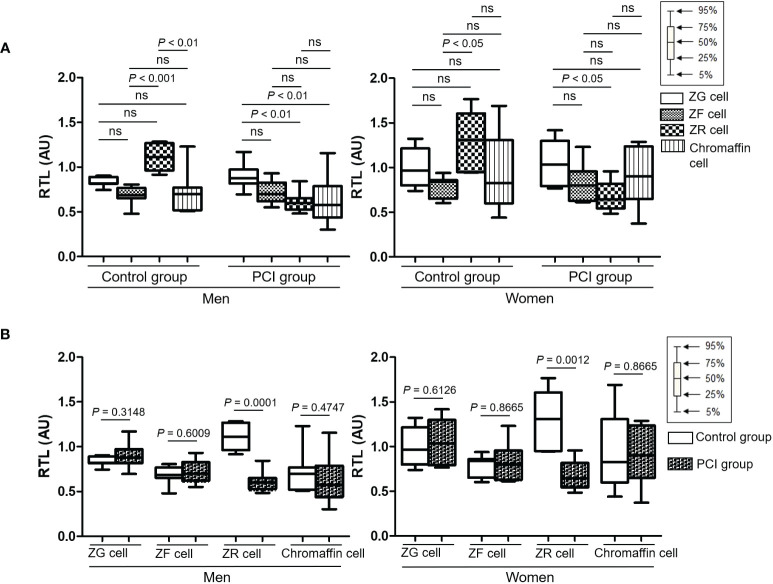
Box plots representing the relative telomere lengths (RTLs) in four types of adrenal parenchymal cells. **(A)** RTLs of different types of adrenal parenchymal cells were compared in the control and prolonged critical illness (PCI) groups. For both men and women, RTLs in ZR cells were the longest in the control group, whereas RTLs in ZR cells were the shortest in the PCI group. **(B)** RTLs of the same type of adrenal parenchymal cells were compared between the control and PCI groups. RTLs in ZR cells were significantly shorter in the PCI group than in the control group for both men and women. AU, arbitrary units. ns, not significant.

**Figure S1** shows the representative Q-FISH images of the adrenal glands. **Table 2** summarizes the RTLs in the three types of adrenocortical parenchymal and chromaffin cells in the adrenal medulla. In the control group, the RTLs in ZR and ZF cells were the longest and shortest, respectively. In the PCI group, the RTLs in ZR and ZG cells were the shortest and longest, respectively (**Figure 2A**). When we compared the RTLs in the same type of adrenal parenchymal cells between the control and PCI groups, the RTLs in ZR cells were shorter in the PCI group than in the control group for both men and women (*P* = 0.0001 in men; *P* = 0.0012 in women; **Figure 2B**). However, the RTLs in the remaining three types of adrenal parenchymal cells did not differ between the control and PCI groups (**Figure 2B**).

**Table 2:** Summary of the relative telomere lengths in the three types of adrenocortical parenchymal and chromaffin cells in the adrenal medulla.

Control group									
Men					Women				
#	ZG cells	ZF cells	ZR cells	Chromaffin cells	#	ZG cells	ZF cells	ZR cells	Chromaffin cells
1	0.90	0.77	1.27	0.74	1	0.97	0.86	1.32	0.60
2	0.89	0.81	1.28	1.23	2	1.32	0.84	1.77	1.69
3	0.86	0.69	1.01	0.70	3	1.00	0.84	1.27	0.83
4	0.74	0.48	0.96	0.51	4	0.91	0.76	1.31	1.24
5	0.82	0.66	1.17	0.52	5	1.21	0.94	1.61	1.31
6	0.81	0.69	0.92	0.55	6	0.80	0.60	0.95	0.44
7	0.82	0.65	1.11	0.77	7	0.74	0.65	0.95	0.66
Mean ± SD	0.83 ± 0.05	0.68 ± 0.10	1.10 ± 0.14	0.72 ± 0.23	Mean ± SD	0.99 ± 0.20	0.78 ± 0.11	1.31 ± 0.28	0.97 ± 0.42
PCI group									
Men					Women				
#	ZG cells	ZF cells	ZR cells	Chromaffin cells	#	ZG cells	ZF cells	ZR cells	Chromaffin cells
1	0.85	0.63	0.56	0.83	1	1.36	1.23	0.96	1.29
2	0.93	0.80	0.64	0.57	2	1.12	0.81	0.64	1.12
3	0.82	0.68	0.57	0.40	3	1.07	0.96	0.65	1.17
4	0.70	0.60	0.49	0.30	4	0.78	0.63	0.54	0.64
5	0.96	0.83	0.65	1.16	5	0.77	0.63	0.55	0.37
6	1.00	0.83	0.66	0.45	6	1.42	0.94	0.85	0.67
7	1.17	0.93	0.84	0.77	7	0.83	0.61	0.48	0.69
8	0.90	0.68	0.54	0.69	8	1.00	0.79	0.72	1.26
9	0.85	0.71	0.63	0.58	Mean ± SD	1.04 ± 0.23	0.83 ± 0.20	0.67 ± 0.15	0.90 ± 0.32
10	0.81	0.55	0.48	0.45					
Mean ± SD	0.90 ± 0.12	0.72 ± 0.11	0.61 ± 0.10	0.62 ± 0.24					

PCI, Prolonged critical illness; ZF, zona fasciculata; ZG, zona glomerulosa; ZR, zona reticularis; SD, standard deviation.

### Number of ZR parenchymal cells and total number of adrenocortical parenchymal cells

**Figure 3:**
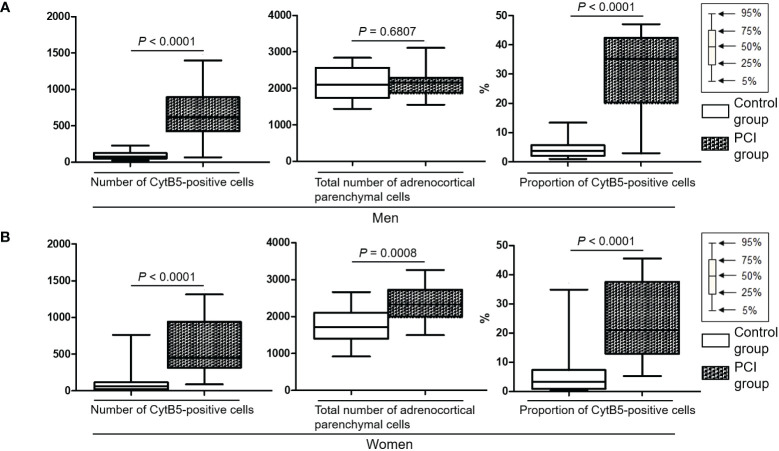
Box plots representing the number of cytochrome B5 (CytB5)-positive cells, total number of adrenocortical parenchymal cells, and proportion of CytB5-positive cells. The proportion of CytB5-positive cells indicates the proportion of the number of CytB5-positive cells to the total number of adrenocortical parenchymal cells. **(A, B)** For both men and women, both the number of CytB5-positive cells and proportion of CytB5-positive cells were significantly higher in the prolonged critical illness (PCI) group than in the control group.

**Figure S2** shows the representative images of double-staining immunohistochemistry for CytB5 and CgA in the adrenal glands. **Figure S3** shows a representative digital image processing of double-staining immunohistochemistry. **Table S1** shows the number of CytB5-positive cells (i.e., ZR parenchymal cells) and the total number of adrenocortical parenchymal cells (i.e., the sum of the numbers of ZG, ZF, and ZR parenchymal cells). In men, the number of CytB5-positive cells was higher in the PCI group than in the control group (*P* < 0.0001), whereas the total number of adrenocortical parenchymal cells did not differ between the two groups (**Figure 3A**). In women, the number of CytB5-positive cells and the total number of adrenocortical parenchymal cells were higher in the PCI group than in the control group (*P* < 0.0001 and *P* = 0.0008) (**Figure 3B**). For both men and women, the proportion of the number of CytB5-positive cells to the total number of adrenocortical parenchymal cells was higher in the PCI group than in the control group (*P* < 0.0001 for both men and women) (**Figures 3A, B**).

### Correlation between disease duration and RTLs in ZR cells in the PCI group

In the PCI group, there was no significant correlation between disease duration (**Table 1**) and RTLs in the ZR cells (**Table 2**) for both men and women (*P* = 0.1231 in men; *P* = 0.6646 in women).

### Correlation between disease duration and number of ZR parenchymal cells in the PCI group

In the PCI group, there was no significant correlation between disease duration (**Table 1**) and the number of CytB5-positive cells (**Table S1**) for both men and women (*P* = 0.5603 in men; *P* = 0.8401 in women).

### Ki-67 proliferation index in ZR parenchymal cells

**Figure S4** shows representative images of double-staining immunohistochemistry for CytB5 and Ki-67 in the adrenal glands. **Table S2** summarizes the number of CytB5-positive cells and Ki-67 proliferation index in CytB5-positive cells. For both men and women, the Ki-67 proliferation index in CytB5-positive cells was higher in the PCI group than in the control group (*P* = 0.0039 in men; *P* = 0.0063 in women).

### Number of adrenocortical cells expressing CYP17A1

**Figure S5** shows representative images of double-staining immunohistochemistry for CYP17A1 and CgA in the adrenal glands. For both men and women, the number of CYP17A1-positive cells was higher in the PCI group than in the control group (*P* < 0.0001 for both men and women) (**Table S3**). Similarly, for both men and women, the proportion of the number of CYP17A1-positive cells to the total number of adrenocortical parenchymal cells was higher in the PCI group than in the control group (*P* < 0.0001 for both men and women) (**Table S3**).

### Antemortem serum DHEA-S levels

DHEA-S comprises the majority (over 90%) of DHEA in circulation owing to its slow rate of clearance from circulation and long biological half-life (35, 36). Thus, we checked the antemortem serum DHEA-S levels instead of DHEA levels. **Table 3** shows a summary of the antemortem serum DHEA-S levels. There were no significant differences in the serum DHEA-S levels between the control and PCI groups for both men and women (*P* = 0.9416 in men; *P* = 0.6905 in women).

**Table 3:** Summary of the antemortem serum DHEA-S levels.

Control group					
Men			Women		
#	DHEA-S (μg/dL)	Collection date of blood samples	#	DHEA-S (μg/dL)	Collection date of blood samples
1	N/A	N/A	1	82	within 24 h before death
2	N/A	N/A	2	66	within 24 h before death
3	287	within 24 h before death	3	127	within 24 h before death
4	16	within 24 h before death	4	N/A	N/A
5	76	within 24 h before death	5	45	within 24 h before death
6	16	within 24 h before death	6	39	within 24 h before death
7	66	within 24 h before death	7	N/A	N/A
PCI group					
Men			Women		
#	DHEA-S (μg/dL)	Collection date of blood samples	#	DHEA-S (μg/dL)	Collection date of blood samples
1	210	3 days before death	1	53	6 days before death
2	146	2 days before death	2	44	1 day before death
3	14	3 days before death	3	67	5 days before death
4	29	1 day before death	4	125	2 days before death
5	17	1 day before death	5	N/A	N/A
6	N/A	N/A	6	210	2 days before death
7	59	4 days before death	7	N/A	N/A
8	68	3 days before death	8	N/A	N/A
9	N/A	N/A			
10	28	1 day before death			

Normal range of serum DHEA-S, 5-253 μg/dL in men aged 71 years and over, 7-177 μg/dL in women aged 71 years and over.

N/A, not applicable; PCI, Prolonged critical illness.

### Antemortem serum albumin levels

**Figure 4:**
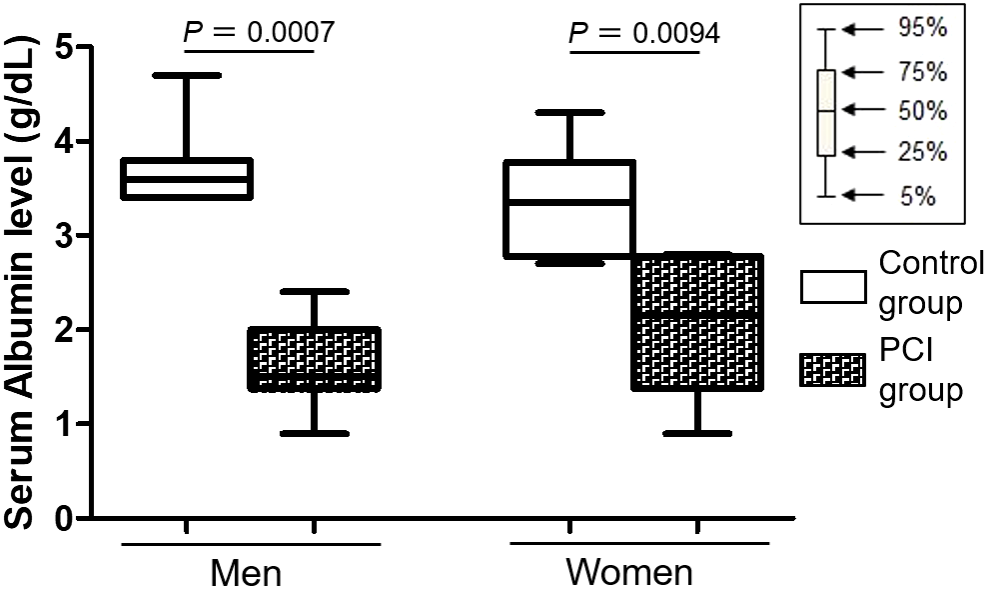
Box plots representing serum albumin levels. Serum albumin levels were lower in the prolonged critical illness (PCI) group than in the control group for both men and women.

The vast majority of DHEA-S in the blood is bound to albumin (37), and changes in the serum albumin levels are related to changes in the serum DHEA-S levels (38). Thus, we checked the antemortem serum albumin levels by referring to the medical records of each patient. **Table 4** shows a summary of the antemortem serum albumin levels at the last laboratory test of a patient’s life. The serum albumin levels were lower in the PCI group than in the control group for both men and women (*P* = 0.0007 in men; *P* = 0.0094 in women) (**Figure 4**). **Figure S6** shows changes in the serum albumin levels during terminal hospitalization in the PCI group.

**Table 4:** Summary of the serum albumin levels on the last laboratory test of the patients.

Control group					
Men			Women		
#	Serum albumin (g/dL)	Date of the last laboratory test	#	Serum albumin (g/dL)	Date of the last laboratory test
1	3.5	32 days before death	1	3.5	within 24 h before death
2	3.4	within 24 h before death	2	2.8	within 24 h before death
3	3.6	within 24 h before death	3	3.6	within 24 h before death
4	3.8	within 24 h before death	4	3.2	within 24 h before death
5	3.8	within 24 h before death	5	2.7	within 24 h before death
6	3.4	within 24 h before death	6	4.3	within 24 h before death
7	4.7	within 24 h before death	7	N/A	N/A
PCI group					
Men			Women		
#	Serum albumin (g/dL)	Date of the last laboratory test	#	Serum albumin (g/dL)	Date of the last laboratory test
1	2.4	3 days before death	1	0.9	within 24 h before death
2	0.9	within 24 h before death	2	1.2	1 day before death
3	1.5	1 day before death	3	1.9	3 days before death
4	1.5	1 day before death	4	2.0	within 24 h before death
5	1.6	within 24 h before death	5	2.3	4 days before death
6	2.0	4 days before death	6	2.8	1 day before death
7	1.4	1 day before death	7	2.8	28 days before death
8	2.0	1 day before death	8	2.7	23 days before death
9	1.4	10 days before death			
10	1.3	within 24 h before death			

N/A, not applicable; PCI, Prolonged critical illness.

### Distribution of oxidative stress and DNA damage in adrenal parenchymal cells

**Figure S7** shows representative images of the immunohistochemistry for 8OHdG and γH2AX in the adrenal glands. In the control group, positive immunoreactivities for both 8OHdG and γH2AX were observed mainly in the ZG and outer ZF for both men and women. However, in the PCI group, positive or weakly positive immunoreactivities for both 8OHdG and γH2AX were diffusely distributed in the adrenal cortex and medulla. Many inflammatory cells also showed positive immunoreactivities for both 8OHdG and γH2AX in the PCI group.

## Discussion

This study showed that in both men and women, the telomere lengths of ZR cells in the PCI group were significantly shorter than those in the control group; however, the mean age of patients in the PCI group was younger than that of patients in the control group by 6.5 years for men and 2.1 years for women. By contrast, the telomere lengths of the remaining three types of adrenal cells (i.e., ZG, ZF, and adrenomedullary chromaffin cells) did not differ between the control and PCI groups, indicating that ZR cell-specific telomere shortening occurred in patients with PCI.

Telomere shortening in human somatic cells largely depends on cell divisions both *in vitro* and *in vivo* (12, 39). In the present study, the number of ZR cells was significantly higher in the PCI group than in the control group for both men and women. Furthermore, the Ki-67 proliferation index in CytB5-positive cells (i.e., ZR parenchymal cells) was significantly higher in the PCI group than in the control group for both men and women, suggesting that reactive proliferation of ZR cells occurred in patients with PCI. Numerous studies have demonstrated that oxidative stress and DNA damage are associated with telomere shortening (40, 41). In the PCI group, however, positive immunoreactivities for both 8OHdG and γH2AX (i.e., representative markers for oxidative stress and DNA damage) were diffusely distributed in the adrenal cortex and medulla, but not localized to the ZR. These findings suggest that the reactive proliferation of ZR cells, rather than oxidative stress and DNA damage, contributes to ZR cell-specific telomere shortening.

Chronic disease burden is reportedly associated with shorter telomere lengths in human tissues (9, 10). The duration of chronic diseases is usually three months or more (42). However, in this study, the disease duration of many patients in the PCI group was less than three months. In addition, the disease duration in the PCI group did not correlate with the telomere lengths of ZR cells or the number of ZR cells. As mentioned previously, this study defined the PCI group on the basis of the presence of adrenal lipid depletion but not on the basis of disease duration. Therefore, these findings suggest that in patients with PCI, ZR cell-specific telomere shortening is more strongly associated with adrenal lipid depletion than with disease duration.

Hypoalbuminemia (< 3.0 g/dL) is a common complication in patients with chronic diseases (43). In the present study, the serum albumin levels in the last laboratory test were significantly lower in the PCI group than in the control group for both men and women. Furthermore, all patients in the PCI group showed hypoalbuminemia during terminal hospitalization. Approximately 95% of serum DHEA-S is bound to albumin (37); therefore, hypoalbuminemia or elevated urinary albumin excretion is associated with low serum DHEA-S levels (44, 45). In this study, however, the serum DHEA-S levels in the PCI group were either within the normal range or higher than the normal levels and were not significantly lower than those in the control group. Furthermore, the number of CYP17A1-positive cells was higher in the PCI group than in the control group. CYP17A1, located in the ZF and ZR parenchymal cells, catalyzes the synthesis of DHEA (30), and the expansion of the ZR correlates with increased production of DHEA-S during childhood (30). Therefore, these findings suggest that the expansion of the ZR with an increased number of CYP17A1-positive cells in the PCI group contributes to the maintenance of serum DHEA-S levels in the presence of hypoalbuminemia.

DHEA and DHEA-S are the most abundant steroid hormones in humans (35). Animal studies in rodents have suggested that DHEA is multifunctional, with immunoenhancing, antidiabetic, anti-obesity, and anti-aging effects (46, 47); however, the beneficial effects of DHEA in humans have not been established (48). The increase in the number of CytB5- and CYP17A1-positive cells observed in the current study suggests that DHEA and DHEA-S are important for bodily functions during PCI.

This study had some limitations typically associated with autopsy studies. First, the sample size of the adrenal specimens was relatively small, mainly because of the difficulty in obtaining appropriate adrenal glands in both the control and PCI groups. Second, we could not retrospectively analyze the changes in the serum levels of DHEA-S and other hormones such as cortisol and adrenocorticotropic hormone because, unlike albumin levels, these hormone levels were not routinely measured.

In conclusion, this study demonstrated ZR cell-specific telomere shortening in patients with adrenal lipid depletion who died after PCI. Our results suggest that the reactive proliferation of ZR cells accelerates the telomere shortening and aging process in ZR cells in these patients. Although the effects of DHEA and DHEA-S in humans are not well understood, the increased number of CytB5- and CYP17A1-positive cells in patients with PCI suggests that these hormones have beneficial effects on the bodily functions of these patients. The results of our study may contribute to the understanding of adrenal aging during PCI. Further large-scale studies are required to validate these findings.

## Data availability statement

The original contributions presented in the study are included in the article/**Supplementary Material**. Further inquiries can be directed to the corresponding author.

## Ethics statement

The studies involving humans were approved by the Ethics Committee of the Tokyo Metropolitan Institute for Geriatrics and Gerontology. The studies were conducted in accordance with the local legislation and institutional requirements. The participants provided their written informed consent to participate in this study.

## Author contributions

Conceptualization: KN, TI, HS and TA. Methodology: KT, JA, YW and TI. Investigation: KN and YW. Formal analysis: KN, YW and TI. Writing – original draft: KN. Writing – review & editing: KN, KT, JA, YW, FG, YS, AK, YY, TI, HS and TA. Resources: KN, JA, AK and TA. Funding acquisition: KN. All authors contributed to the article and approved the submitted version.
