# Supplementary material for: Accelerated telomere shortening in adrenal zona reticularis in patients with prolonged critical illness
Source: Front Endocrinol (Lausanne). 2023 Sep 4;14:1244553. doi: 10.3389/fendo.2023.1244553 (PMC10512174; doi:10.3389/fendo.2023.1244553)
Supplement: Supplementary file 1 [file DataSheet_1.docx]

Supplementary Material

Accelerated telomere shortening in adrenal zona reticularis in patients with prolonged critical illness

Keisuke Nonaka^*^, Kaiyo Takubo, Junko Aida, Yoriko Watai, Akiko Komatsu, Fujiya Gomi, Yuuki Shichi, Yuto Yamazaki, Toshiyuki Ishiwata, Hironobu Sasano, Tomio Arai

*** Correspondence:** Keisuke Nonaka: nona_kei@tmig.or.jp

# Supplementary Figures and Tables

## Supplementary Figures


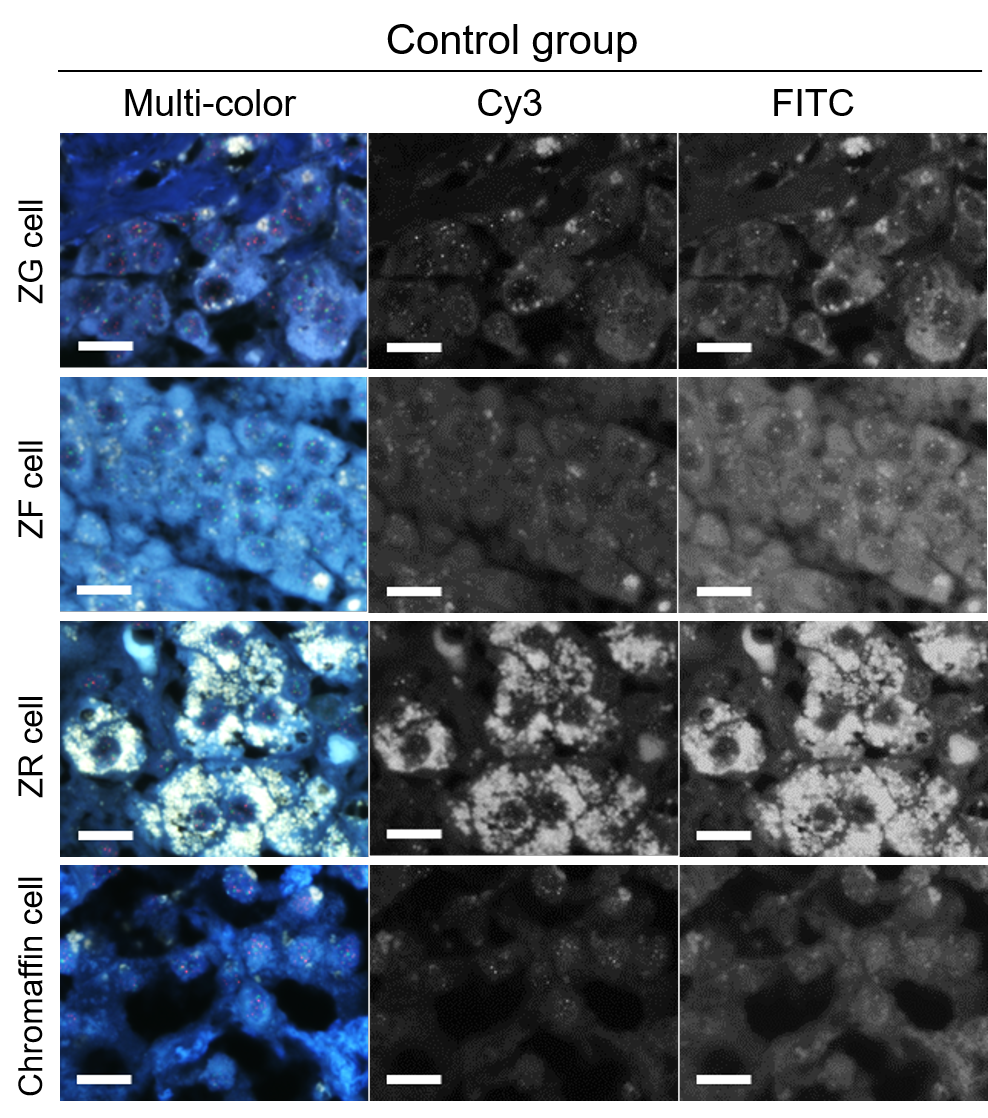


(A)


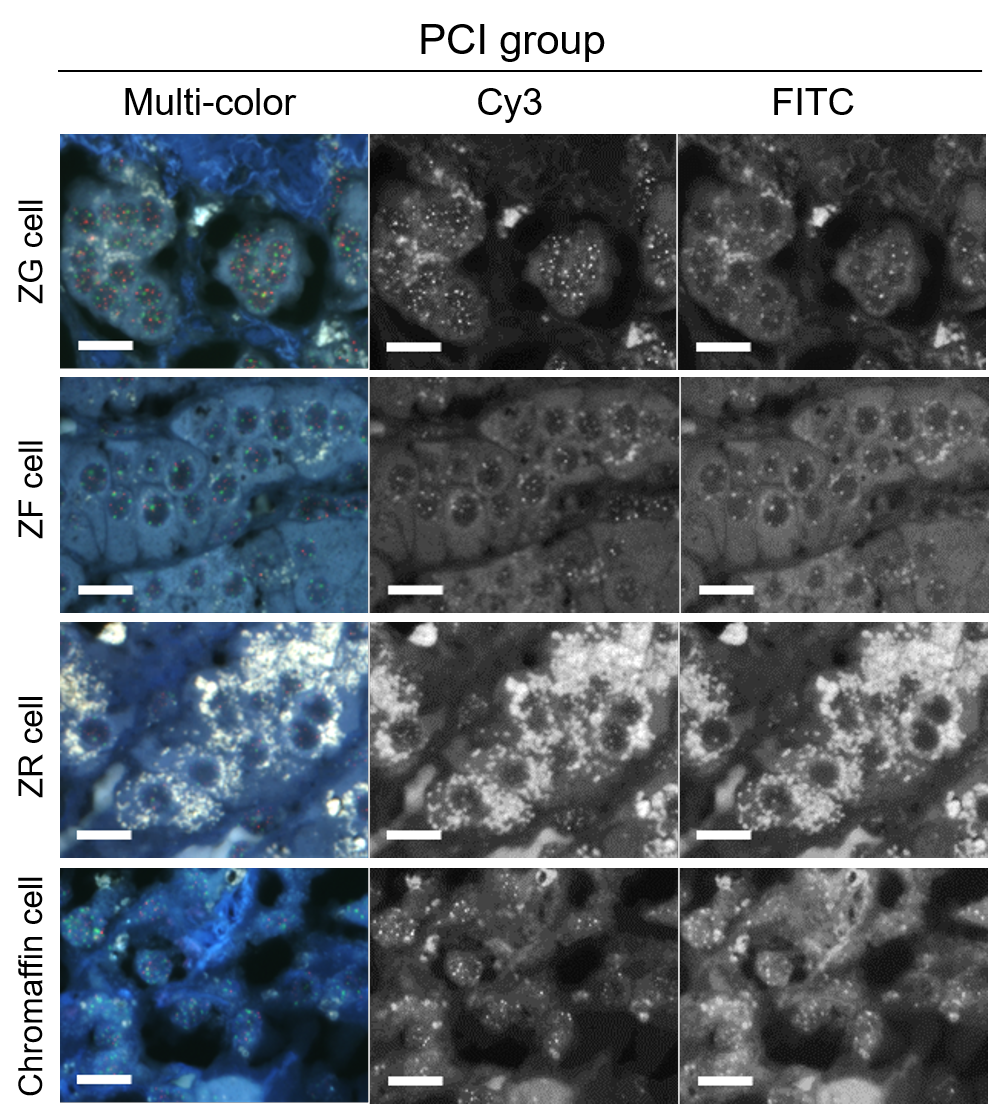


(B)

**Supplementary Figure 1.** Representative quantitative fluorescence *in situ* hybridization (Q-FISH) images of the adrenal gland. **(A)** An 81-year-old man in the control group. **(B)** An 81-year-old man in the prolonged critical illness (PCI) group. **(A and B)** In the multi-color images, the red (Cy3) and green (fluorescein isothiocyanate, FITC) colors indicate the telomere and centromere signals, respectively. In patients from both the control and PCI groups, zona glomerulosa (ZG) parenchymal cells have less abundant cytoplasm and are located near the adrenal capsule, whereas zona fasciculata (ZF) parenchymal cells are organized into bundles. Zona reticularis (ZR) parenchymal cells can be identified because their cytoplasm has prominent lipofuscin and appear as brilliant yellow autofluorescence in Q-FISH images. Chromaffin cells in the medulla have relatively regular nuclei in terms of size and contour. Scale bar in **(A)** and **(B)**, 10 μm.


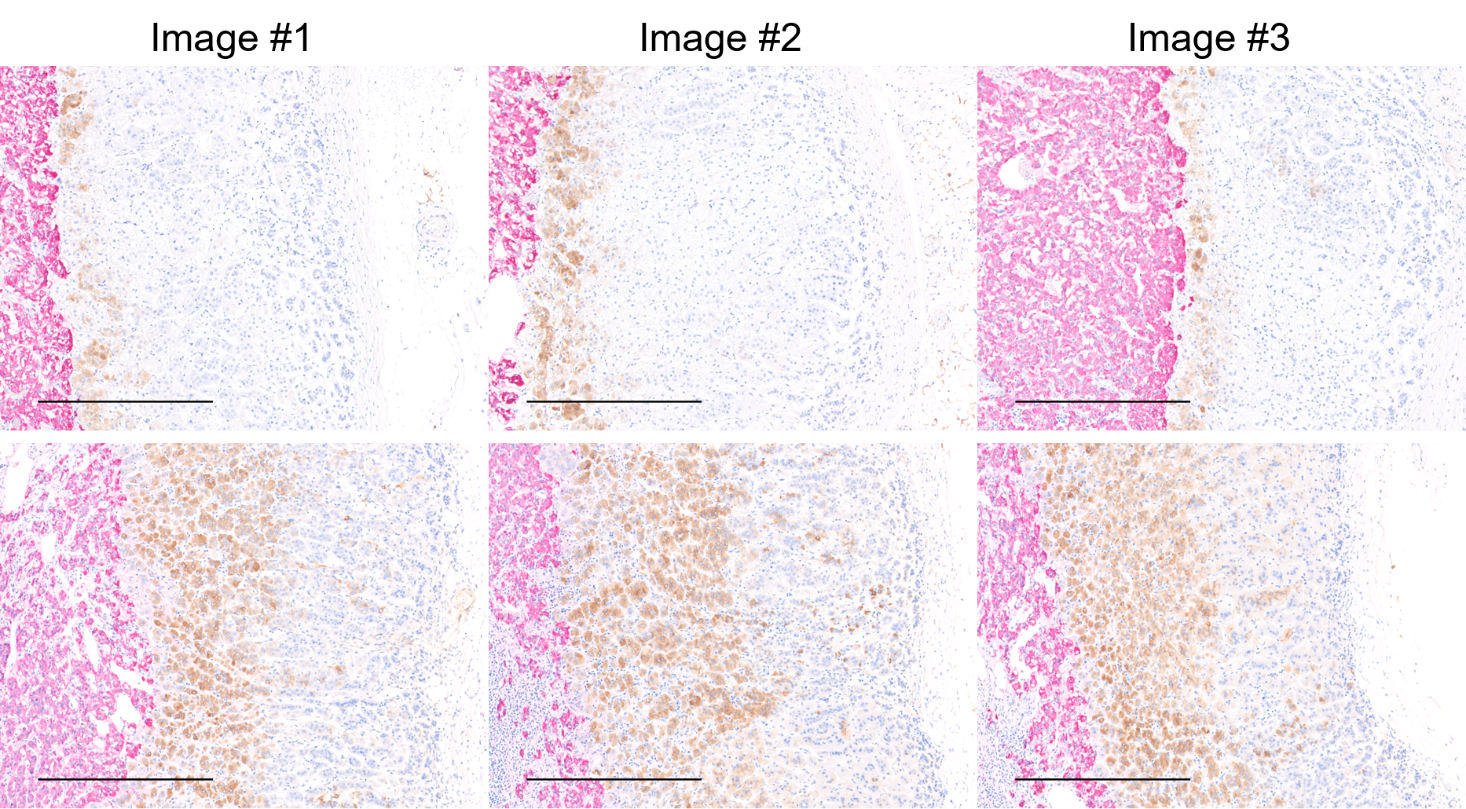


(B)

(A)

**Supplementary Figure 2.** Representative images of double-staining immunohistochemistry for cytochrome B5 (CytB5) and chromogranin A (CgA) of the adrenal gland. **(A)** An 85-year-old woman in the control group. **(B)** An 87-year-old woman in the prolonged critical illness group. **(A and B)** For each patient, three arbitrary digital images (Images #1–#3) of immunohistochemically stained sections were taken. Each image covered the area from the adrenal capsule to the medulla. 3,3′-Diaminobenzidine (DAB) staining represents CytB5-positive cells (i.e., zona reticularis parenchymal cells). Red staining represents CgA-positive cells (i.e., adrenomedullary chromaffin cells). Scale bar in **(A)** and **(B)**, 500 μm.

(B)

(A)

**
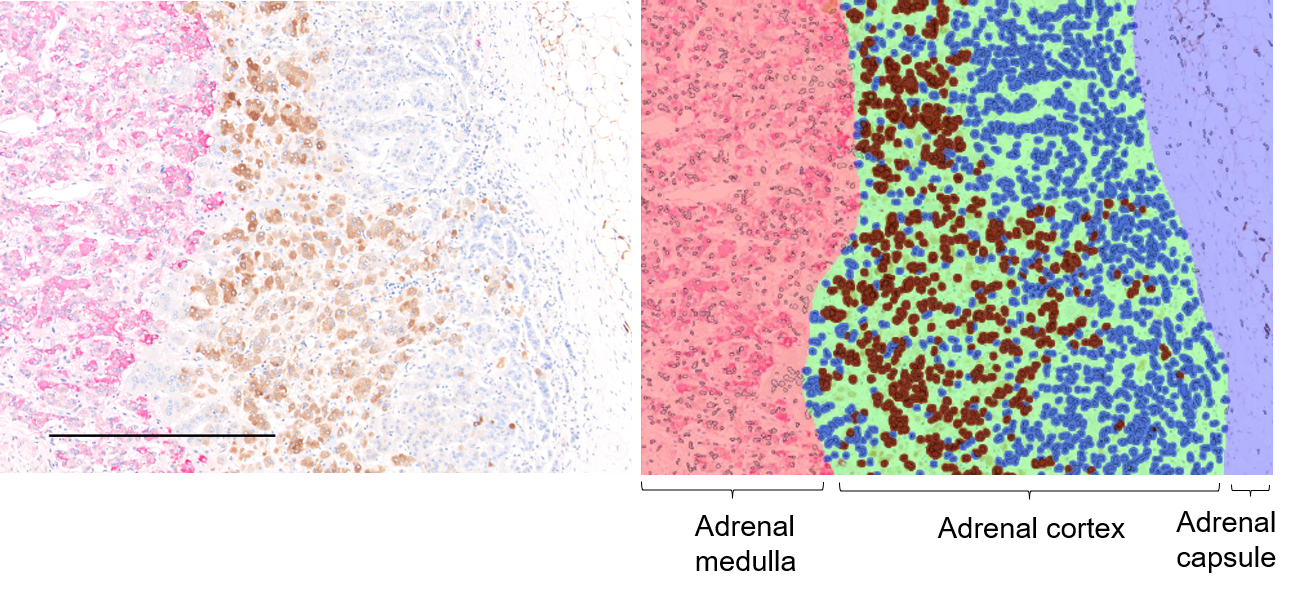
**

**Supplementary Figure 3.** Representative digital image processing of the double-staining immunohistochemistry. **(A)** Double-staining immunohistochemistry for cytochrome B5 (CytB5) and chromogranin A (CgA) of the adrenal gland. 3,3′-Diaminobenzidine (DAB) staining represents CytB5-positive cells (i.e., zona reticularis parenchymal cells). Red staining represents CgA-positive cells (i.e., adrenomedullary chromaffin cells). Red staining is helpful for the machine-learning algorithms to distinguish adrenocortical parenchymal cells from adrenomedullary chromaffin cells. Scale bar, 500 μm. **(B)** In the captured image, the green-colored area represents the adrenal cortex, the brown-colored dots represent CytB5-positive cells, the blue-colored dots represent CytB5-negative adrenocortical parenchymal cells (i.e., a mixed population of zona glomerulosa and zona fasciculata parenchymal cells), and the red-colored area represents the adrenal medulla.


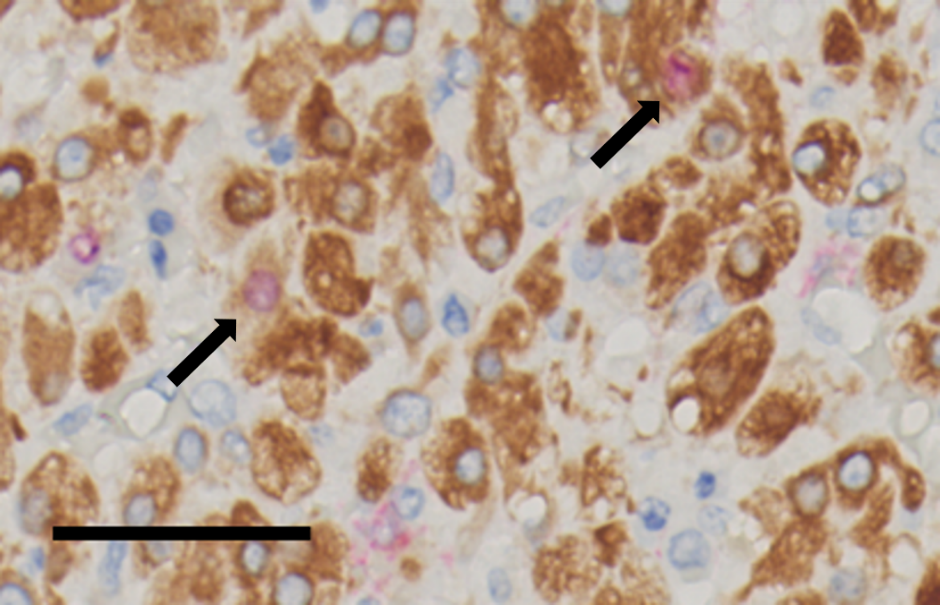


**Supplementary Figure 4.** Representative image of double-staining immunohistochemistry for cytochrome B5 (CytB5) and Ki-67 of the adrenal gland. A 77-year-old woman in the prolonged critical illness group. 3,3′-Diaminobenzidine (DAB) cytoplasmic staining represents CytB5-positive cells (i.e., zona reticularis parenchymal cells). Red nuclear staining represents Ki-67-positive cells (arrows). Scale bar, 50 μm.


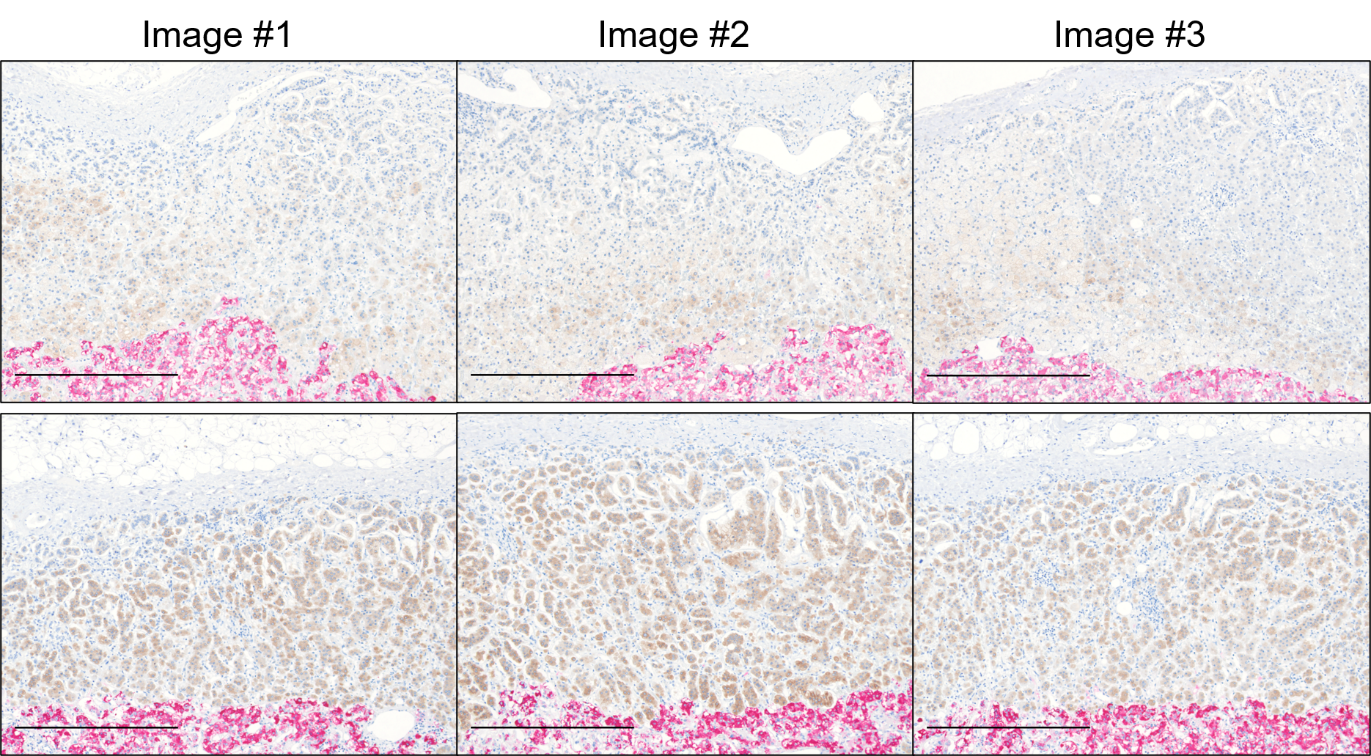


(B)

(A)

**Supplementary Figure 5.** Representative images of double-staining immunohistochemistry for cytochrome P450 17A1 (CYP17A1) and chromogranin A (CgA) of the adrenal gland. (A) An 89-year-old man in the control group. (B) A 73-year-old man in the prolonged critical illness group. (A and B) For each patient, three arbitrary digital images (Images #1–#3) of immunohistochemically stained sections were taken. Each image covered the area from the adrenal capsule to the medulla. 3,3′-Diaminobenzidine (DAB) staining represents CYP17A1-positive cells. Red staining represents CgA-positive cells (i.e., adrenomedullary chromaffin cells). Scale bar in (A) and (B), 500 μm.

**
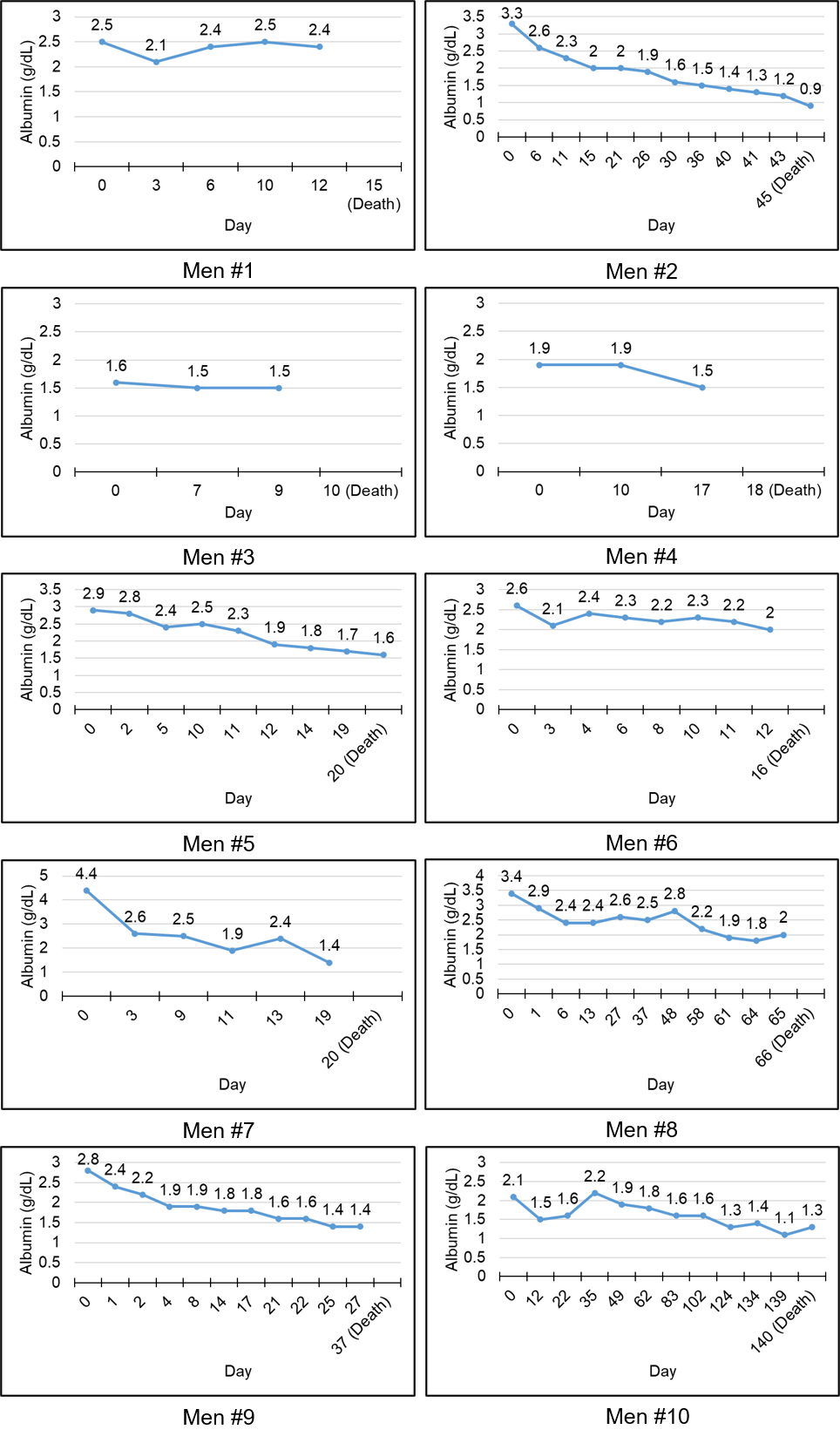
**

(A)

**
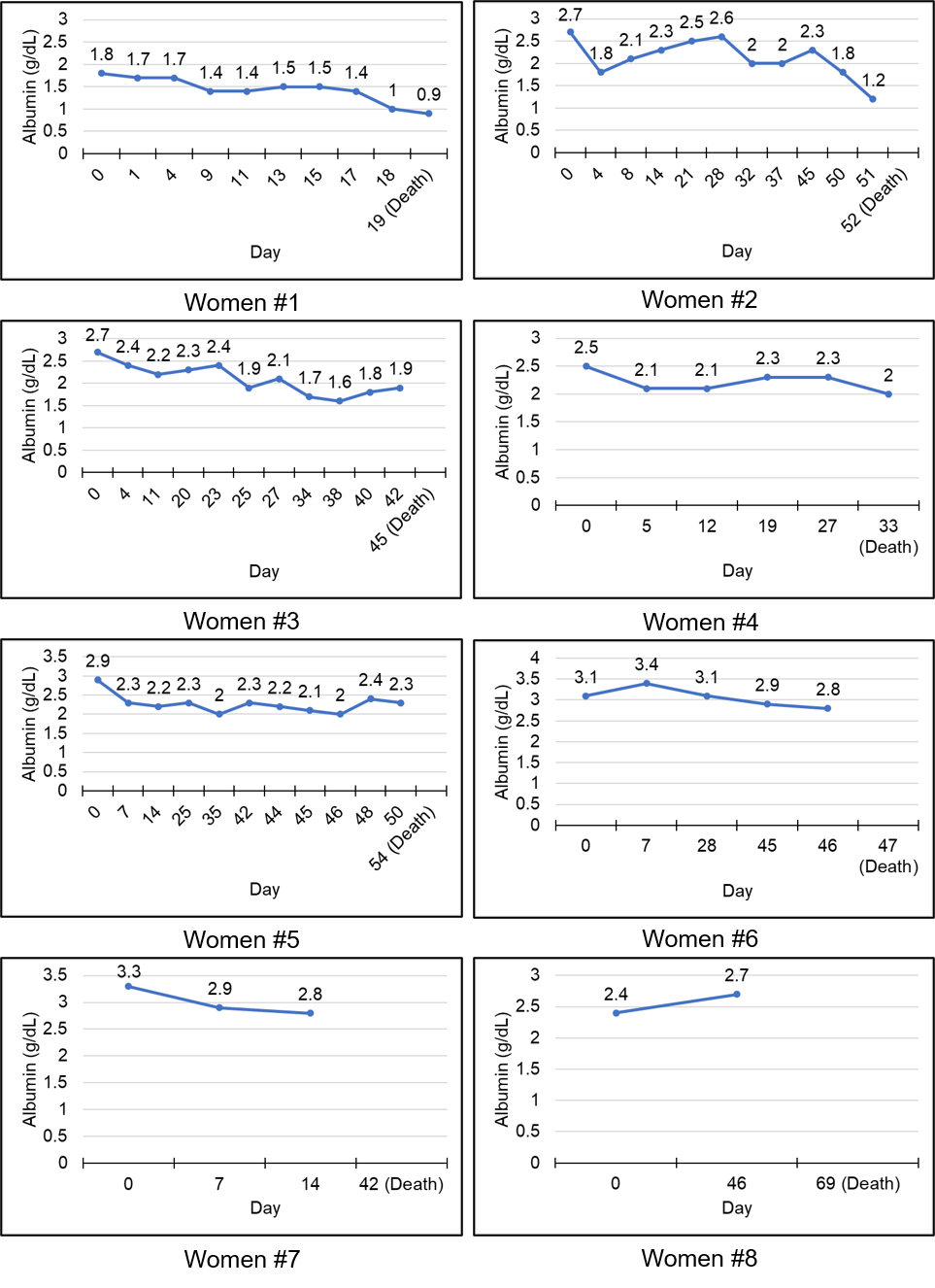
**

(B)

**Supplementary Figure 6.** Changes in serum albumin levels during terminal hospitalizations in the prolonged critical illness group. **(A)** Men #1–#10. **(B)** Women #1–#8.


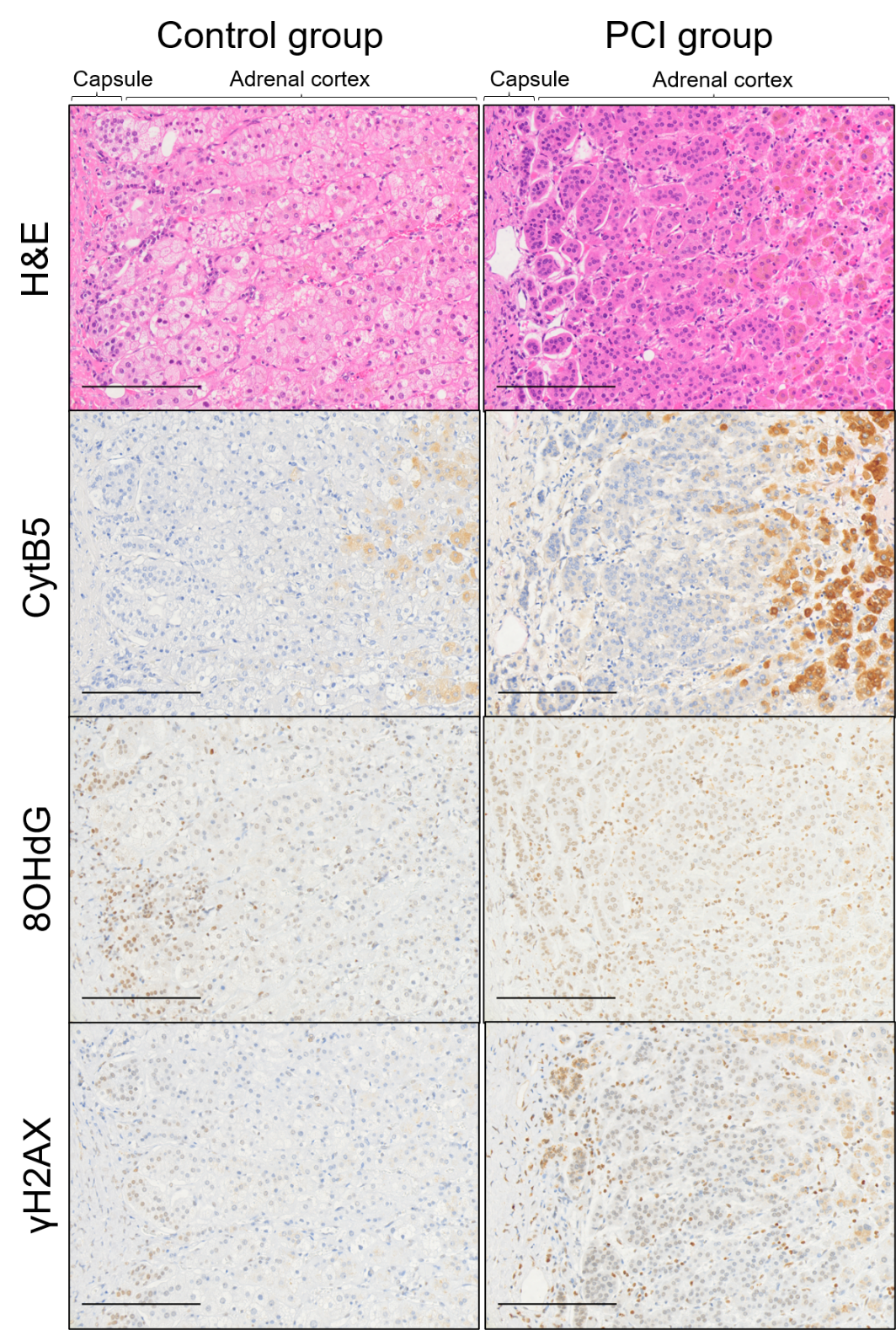


**Supplementary Figure 7.** Representative images of hematoxylin and eosin (H&E) staining and immunohistochemistry for cytochrome B5 (CytB5), 8-hydroxydeoxyguanosine (8OHdG), and phosphorylated gamma histone H2AX (γH2AX). CytB5-positive cells represent zona reticularis parenchymal cells. In patients from the control group, positive immunoreactivities for both 8OHdG and γH2AX were seen mainly in the zona glomerulosa and outer zona fasciculata. In patients from the prolonged critical illness (PCI) group, positive or weakly positive immunoreactivities for both 8OHdG and γH2AX were diffusely distributed in the adrenal cortex. Scale bar, 200 μm.

## Supplementary Tables

**Supplementary Table 1.** Summary of the number of cytochrome B5 (CytB5)–positive cells, total number of adrenocortical parenchymal cells, and proportion of CytB5-positive cells

**Supplementary Table 1.** Continued

Proportion of CytB5-positive cells indicates the proportion of the number of CytB5-positive cells to the total number of adrenocortical parenchymal cells. Abbreviations: PCI, Prolonged critical illness; SD, standard deviation.

**Supplementary Table 2.** Summary of the number of cytochrome B5 (CytB5)-positive cells and Ki-67 proliferation index in CytB5-positive cells

**Supplementary Table 2.** Continued

Abbreviations: CytB5, Cytochrome B5; PCI, Prolonged critical illness; SD, standard deviation.

**Supplementary Table 3.** Summary of the number of cytochrome P450 17A1 (CYP17A1)-positive cells and proportion of CYP17A1-positive cells

**Supplementary Table 3.** Continued

Proportion of CYP17A1-positive cells indicates the proportion of the number of CYP17A1-positive cells to the total number of adrenocortical parenchymal cells. Abbreviations: PCI, Prolonged critical illness; SD, standard deviation.
